# Fueling the Cycle: CDKs in Carbon and Energy Metabolism

**DOI:** 10.3389/fcell.2018.00093

**Published:** 2018-08-17

**Authors:** Maria Solaki, Jennifer C. Ewald

**Affiliations:** Interfaculty Institute of Cell Biology, Eberhard Karls University of Tuebingen, Tuebingen, Germany

**Keywords:** cell division cycle, proliferation, cyclin-dependent kinase, metabolism, energy homeostasis, metabolic fluxes, signaling

## Abstract

Cyclin-dependent kinases (CDKs) are the central regulators of the eukaryotic cell cycle, and are conserved across eukaryotes. Their main and well-studied function lies in the regulation and the time-keeping of cell cycle entry and progression. Additionally, more and more non canonical functions of CDKs are being uncovered. One fairly recently discovered role of CDKs is the coordination of carbon and energy metabolism with proliferation. Evidence from different model organisms is accumulating that CDKs can directly and indirectly control fluxes through metabolism, for example by phosphorylating metabolic enzymes. In this mini-review, we summarize the emerging role of CDKs in regulating carbon and energy metabolism and discuss examples in different models from yeast to cancer cells.

## Introduction

The eukaryotic cell division cycle is a series of tightly coordinated processes that lead to duplication of DNA and accurate distribution of the genetic material into two daughter cells. The most important regulators driving and coordinating the cell cycle are the cyclin dependent kinases (CDKs) (Morgan, 2007, 2008). CDKs are well conserved between eukaryotes. Though the number of different isoforms varies between species, their structure and function is very similar (Malumbres, 2014): The cell cycle kinases themselves are usually present in constant concentrations, while their different regulatory subunits, the cyclins, are expressed only in specific phases of the cell cycle and thereby drive the cell cycle clock forward.

**Figure 1:**
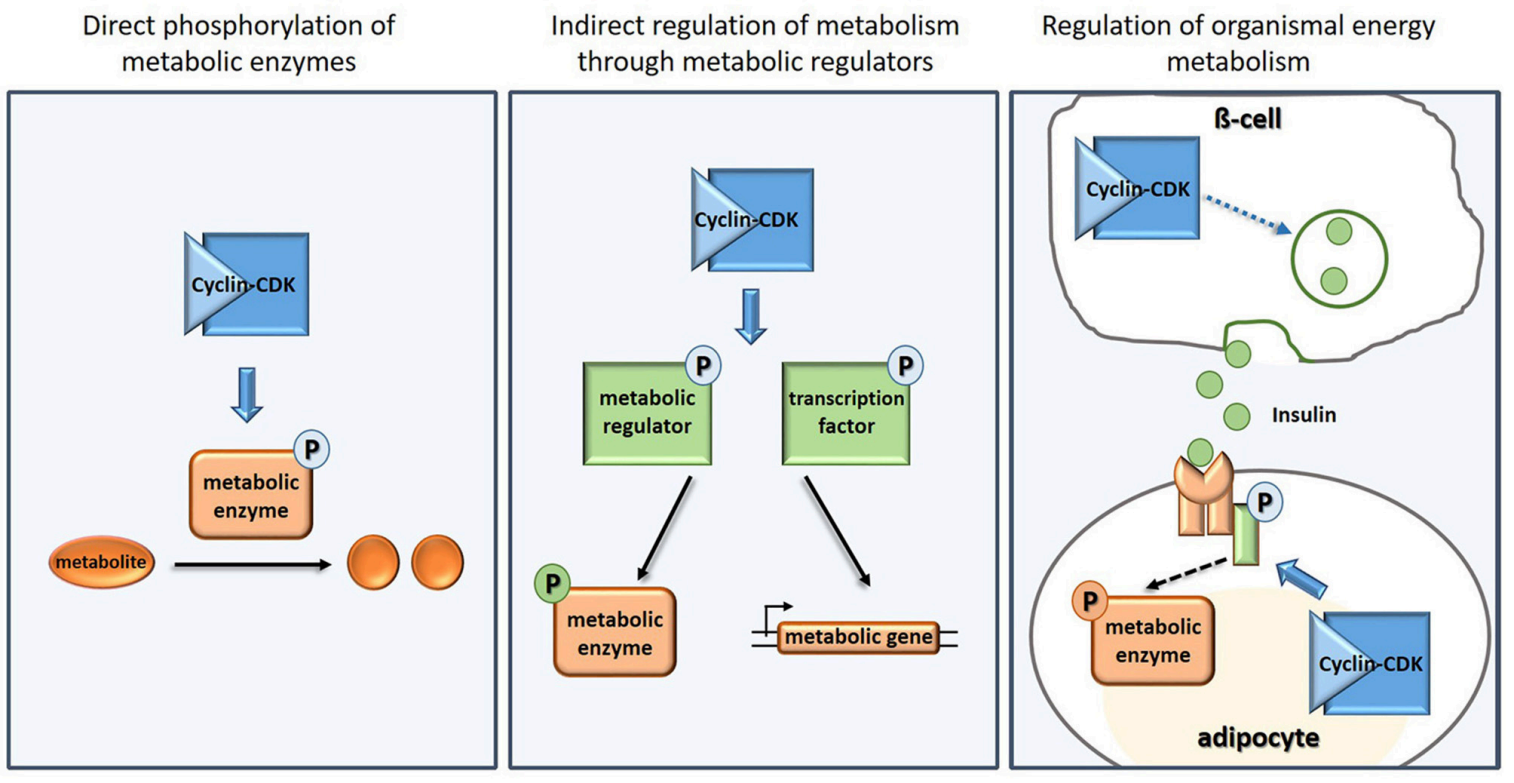
Examples of how CDKs regulate carbon and energy metabolism directly **(Left)**, indirectly **(Middle)**, and on organismal level **(Right)**.

To drive cell cycle progression, CDKs phosphorylate many other cell cycle executers and regulators such as the cell cycle inhibitors Whi5 (de Bruin et al., 2004) and pRb (Lees et al., 1991), the origin recognition complex (Dahmann et al., 1995), or the anaphase-promoting complex (Rudner and Murray, 2000). However, when researchers started systematic and unbiased CDK targets screens (Ubersax et al., 2003; Chi et al., 2008; Dephoure et al., 2008; Holt et al., 2009; Errico et al., 2010), more and more targets not directly associated with the cell cycle were revealed. Many of these targets are involved in processes generally associated with cellular “house-keeping” such as translation, trafficking, and metabolism. The targets in metabolism include metabolic enzymes, metabolic regulators and molecules involved in organismal energy homeostasis (Figure 1, Table 1, Supplementary Table 1).

**Table 1:** Examples of cell cycle CDKs regulating carbon and energy metabolism.

**Publication**	**Organism**	**Cyclin-CDK**	**Metabolic Target**	**Cell cycle dependent?**	**Metabolic Effect**
**DIRECT PHOSPHORYLATION OF METABOLIC ENZYMES**					
Wang et al., 2014	*Homo sapiens*/MCF-10A cells	cyclin B1-CDK1	CI subunits of the respiratory chain	Yes	Increase of mitochondrial respiration
Ewald et al., 2016	*Saccharomyces cerevisiae*	CDK1	Nth1	Yes	Liquidation of trehalose/increase of internal glucose supply
Zhao et al., 2016	*Saccharomyces cerevisiae*	CDK1	Nth1, Gph1	Yes	Liquidation of storage carbohydrates/increase of internal glucose supply
Harashima et al., 2016	*Arabidopsis thaliana*	CDKA;1	mMDH1, ALDH7B4, pfkB-like kinase, IMD1	Unclear	Not determined
Wang et al., 2017	*Homo sapiens*/T-ALL cell lines	cyclin D3-CDK6	PFK1, PKM2	Unclear	Reprogramming metabolism toward PPP and serine pathway/detox ROS
**INDIRECT REGULATION OF METABOLISM**					
Wang et al., 2006	*Mus musculus*	cyclin D-CDK	NRF1	Unclear	Inhibition of mitobiogenesis
Icreverzi et al., 2012	*Drosophila melanogaster*	cyclin D-CDK4	NRF-1 targets	Unclear	Regulation of mitobiogenesis
Harbauer et al., 2014	*Saccharomyces cerevisiae*	Clb3- CDK1	Tom6	Yes	Increase in mitochondrial respiration
Lopez-Mejia et al., 2017	*Mus musculus*/MEFs *Mus musculus/*C57BL6	CDK4	AMPKα2	Unclear	Upregulation of glycolysis, inhibition of fatty acid oxidation
**REGULATION OF ORGANISMAL ENERGY METABOLISM**					
Annicotte et al., 2009	*Mus musculus* /Min6 cells	CDK4 (-pRb-E2F)	Kir6.2	Unclear	Insulin secretion
Lee et al., 2014	*Homo sapiens*/U-2OS cells *Mus musculus* /C57BL6	cyclin D1-CDK4	GCN5	No	Suppression of hepatic gluconeogenesis
Lagarrigue et al., 2016	*Mus musculus/* C57BL6	cyclin D3-CDK4	IRS2	Unclear	Maintenance of insulin signaling in adipocytes
Kim et al., 2017	*Mus musculus*	CDK2	FOXO1	Unclear	Regulation of β-cell mass and β-cell function

The identification of metabolic targets of CDK highlights the tight links between metabolism, growth and the cell cycle, which are being increasingly appreciated in basic and clinical research (Vander Heiden et al., 2009; Galluzzi et al., 2013; Salazar-Roa and Malumbres, 2017; Ewald, 2018). The cell cycle is a series of macromolecular events, each having specific demands for anabolic precursors and energy. In this mini-review, we summarize the role of CDKs in meeting these metabolic demands by regulating carbon and energy metabolism in different organisms (Table 1).

## CDKs directly regulating metabolic enzymes

As cells progress through the cell cycle, CDKs phosphorylate downstream regulators and proteins directly involved in executing DNA synthesis and cell division (Morgan, 2008). Since the discovery of CDKs, their target list is continually growing. The first mid-size screen searching for CDK substrates in the yeast *S. cerevisiae* was presented by Ubersax et al. (2003). This work defined a list of 181 proteins that were phosphorylated by CDK in extracts; many of these targets were not previously associated with the cell cycle, including several enzymes in carbohydrate and lipid metabolism. Two enzymes from lipid metabolism found in this list were later verified as CDK targets and were shown to be regulated in their activity by CDK (Santos-Rosa et al., 2005; Kurat et al., 2009).

The first large-scale untargeted phosphoproteomics experiments about a decade ago investigated the cell cycle and massively expanded the list of likely CDK targets. These studies revealed that neither the change of phosphorylation during the cell cycle, nor the list of direct CDK targets was limited to proteins generally considered to participate in the core cell cycle (Chi et al., 2008; Dephoure et al., 2008; Holt et al., 2009; Carpy et al., 2014). In fact, the list of CDK targets from Holt et al. (2009) was later re-analyzed with respect to metabolic targets (Zhao et al., 2016): Over a third of the 309 CDK targets fall into the broad category “metabolism”; at least a dozen of these are enzymes catalyzing reactions in central carbon, energy, or lipid metabolism.

Based on this initial evidence, two labs recently independently showed that yeast carbohydrate metabolism is regulated by CDK1 (the only cell cycle CDK in yeast) in a cell cycle dependent manner (Ewald et al., 2016; Zhao et al., 2016; Figure 1, Table 1). The enzymes Nth1 and Gph1 are activated by CDK to liquidate the carbohydrate storage molecules trehalose and glycogen, thereby generating glucose. This regulation is especially important in nutrient-limited environments, when cells are faced with sudden nutrient depletion (Ewald et al., 2016) or are approaching stationary phase (Zhao et al., 2016). Thus, CDK directly controls the increase of glycolytic flux at the G1/S transition to ensure sufficient carbon and energy supply during the yeast cell cycle.

A recent study in human cells also shows how CDK can control glycolytic flux (Wang et al., 2017), albeit in a different context. Wang et al. showed an interaction of CDK6-Cyclin D3 (G1/Interphase CDK in mammals) with nine out of eleven glycolytic enzymes in human cancer cells (Wang et al., 2017). The authors functionally characterized two of these enzymes, PFKP and PKM2. These enzymes catalyze the reaction of glucose-6-phosphate to fructose-bis-phosphate and the reaction from phospho-enol-pyruvate to pyruvate, respectively. PFKP and PKM are not only rate-controlling enzymes of glycolysis, but are also well known in the context of cancer (Al Hasawi et al., 2014; Lunt et al., 2015; Webb et al., 2016; Hsu and Hung, 2018). Both phosphorylations described in this study inhibit catalytic activity of the respective enzymes, presumably to re-direct flux from glycolysis into the pentose-phosphate-pathway and serine pathways to promote anabolism and antioxidant metabolism (Wang et al., 2017). Preventing flux into the pentose-phosphate-pathway in these cells led to depletion of antioxidants and a reactive oxygen induced apoptosis.

It remains to be shown whether this regulation is also physiologically relevant in healthy proliferating cells, and whether the CDK6 dependent catalytic activity of PFKP and PKM oscillates with the cell cycle. This is especially interesting since both the fructose-bis-phosphate and the pyruvate node are known to be regulated by multiple mechanisms, including other cell cycle regulators. For example, the ubiquitin ligases APC and SCF, both important cell cycle regulators, control the concentration of PFKFB3, which in turn produces fructose-2,6-bisphosphate, an activator of PFKP (Almeida et al., 2010; Tudzarova et al., 2011).

While the regulation of glycolysis and pentose-phosphate fluxes seems to be especially important for passing the restriction point and progressing through S-phase, mitochondrial metabolism takes center stage during mitosis (Bao et al., 2013; Harbauer et al., 2014). Consistent with the idea that mitochondrial ATP production is important for mitosis, CDK regulates mitochondrial metabolism both indirectly (see below) and directly in late cell cycle stages. Wang et al. showed in human cell lines that CDK1-Cyclin B1, which is important for entering and executing mitosis, partially localizes to the mitochondrial matrix (Wang et al., 2014). There, it phosphorylates several proteins of complex I of the respiratory chain. This leads to an activation of respiration and increased mitochondrial ATP production, which seems to be required for timely completing mitosis (Wang et al., 2014).

The three abovementioned mechanisms—regulation of glycolytic flux by the APC, diversion of intermediates into the pentose-phosphate-pathway through CDK regulation, and activation of mitochondrial respiration by CDK—may lead to cycling of carbon metabolism during the cell cycle to balance anabolic and energy fluxes: glycolytic flux is increasing throughout G1, nucleotide precursors for RNA and DNA metabolism are provided during late G1 and S phase, and mitochondrial ATP production is enhanced during mitosis. However, other studies have reported maximal respiration during late G1 phase (Schieke et al., 2008; Mitra et al., 2009), indicating there may be cell type or tissue specific regulatory mechanisms.

In plants, CDK also seems to target central carbon metabolism. A study in *Arabidopsis thaliana* recently identified several enzymes of glycolysis and mitochondrial metabolism as direct CDK-A (the CDK that drives G1/S/G2 in plants) substrates (Harashima et al., 2016). The function of these sites and their role in the cell cycle has yet to be determined, but this finding suggests that the direct regulation of central carbon metabolism by CDKs is conserved from yeast to mammals to plants.

## CDKs targeting metabolic regulators

In addition to the direct phosphorylation of metabolic enzymes, CDK also targets metabolic pathways indirectly (Figure 1), by phosphorylating regulators of central carbon metabolism, regulators of mitochondria, and transporters.

Both human cells and yeast cells have been observed to increase respiration and mitochondrial ATP production during mitosis (Bao et al., 2013; Harbauer et al., 2014; Wang et al., 2014). However, in budding yeast a direct phosphorylation of respiratory chain components has not been shown as in human cells (see above). In this organism, CDK targets mitochondrial import. Specifically, the phosphorylation of the mitochondrial outer membrane transporter Tom6 enhances its import into the mitochondria, which further supports the assembly of other transporter components (Harbauer et al., 2014). This leads to more import of membrane proteins, including the fusion protein Mgm1. This in turn, like in human cells, leads to an increase in respiration and mitochondrial ATP production during the late phases of the cell cycle (Harbauer et al., 2014).

Not only protein import, but also other aspects of mitochondrial biogenesis (Sakamaki et al., 2006; Wang et al., 2006; Baltzer et al., 2009; Icreverzi et al., 2012) and mitochondrial morphology (Taguchi et al., 2007) have been shown to be regulated by CDK1 (active in G2/M) and CDK4 (active in G1) in flies and mammals. For example, the nuclear respiration factor (NRF1/2), responsible for the transcription of many nuclear-encoded mitochondrial genes, is regulated by CDK4-Cyclin D in both flies (Icreverzi et al., 2012) and mice (Wang et al., 2006). However, the effect of this regulation and the impact on mitochondrial metabolism remains unclear and may be different in each organism. The many additional layers of cross-talk between mitochondrial biogenesis and the cell cycle are extensively discussed in two recent reviews (Lopez-Mejia and Fajas, 2015; Horbay and Bilyy, 2016).

To globally target metabolic fluxes besides the mitochondria, cell cycle regulators might regulate metabolic master regulators such as TOR (target of rapamycin), PKA (protein-kinase A), or AMPK (adenylate-monophosphate activated kinase). Consistent with this idea, the PKA regulatory subunit Bcy1 was shown to be a likely CDK target in two screens in yeast (Ubersax et al., 2003; Holt et al., 2009). However, functional evidence that PKA or other metabolic master regulators are directly controlled by CDK in yeast is (to the best of our knowledge) still lacking. Such evidence was recently found in mouse embryonic fibroblasts: CDK4 directly phosphorylates and thereby inhibits the metabolic master regulator AMPK (specifically only the alpha2 subunit) (Lopez-Mejia et al., 2017). AMPK is the cell's energy barometer. Upon activation this kinase inhibits energy consuming processes and activates catabolism (Lin and Hardie, 2018). Lopez-Meija et al. showed that CDK inhibits AMPK to promote glycolysis and inhibit fatty acid oxidation. This seems to be yet another interesting mechanism by which rapidly proliferating cells can establish a “Warburg-metabolism” (Warburg, 1956) involving aerobic glycolysis, active anabolism and repressed catabolic fluxes (Vander Heiden et al., 2009). It will be interesting to investigate whether this specific inhibition of AMPK is also relevant in CDK4 hyper-activated cancer cells (Qie and Diehl, 2016).

In the same study, the inhibition of AMPK by CDK was further investigated *in vivo* at the organismal level. CDK4 knockout mice increase their oxidative metabolism and exercise capacity in an AMPK dependent manner (Lopez-Mejia et al., 2017), though it remains unclear if and how this effect is linked to growth or the cell cycle. The regulation of AMPK by CDK4 is thus one of the many mechanisms by which CDKs contribute to organismal energy homeostasis, as further detailed in the next section.

## Organismal energy homeostasis—CDKs and insulin signaling

The role of CDKs—both canonical cell cycle, and non-cell cycle CDKs—in regulating organismal energy homeostasis was recognized even before their role in cellular metabolism was uncovered. CDKs act both as targets and as modulators of insulin and growth factor signaling (Figure 1), in proliferating and also in non-proliferating cells. This rather complex topic deserves its own review (see for example Lopez-Mejia et al., 2018); we here thus only provide a brief overview of some examples and emerging patterns.

The G1 kinase CDK4 plays a dual role in insulin signaling, functioning both in insulin-generating cells and in target cells. In mice, CDK4 was observed to regulate insulin secretion in insulin-producing pancreatic β-cells through the CDK4-pRb-E2F1 pathway (Annicotte et al., 2009). In response to high glucose, CDK4 kinase activity is increased through the insulin pathway resulting in pRb phosphorylation. Phosphorylated pRb releases the E2F1-DP complex which, in turn, activates the transcription of the *Kir6.2* gene. Kir6.2 codes for a key component of the K_ATP_ channel involved in insulin secretion. CDK4, pRb and E2F1 expression was observed in nearly all insulin-producing β-cells, even though they were predominantly not proliferating (Annicotte et al., 2009). However, it remains unclear whether this pathway functions fully independently of the cell cycle in regulating insulin secretion.

Besides its role in insulin-generating β-cells, CDK4 was also described to act in insulin target cells such as hepatocytes and adipocytes (Lee et al., 2014; Lagarrigue et al., 2016). In these cells CDK is both a target and modulator of insulin signaling. For example, insulin activates hepatic cyclin D1-CDK4 which, in turn, phosphorylates the acetyltransferase GCN5. GCN5 subsequently acetylates PGC-1α thereby inhibiting the expression of gluconeogenic genes. This pathway involving cyclin D1-CDK4 is responsible for controlling glucose metabolism by suppressing hepatic glucose production in mice. Despite the activation of cyclin D1-CDK4 by insulin, the hepatocytes were not proliferating, indicating that the regulation of hepatic gluconeogenesis by cyclin D1-CDK4 is not cell cycle dependent (Lee et al., 2014). Other target cells of insulin, in which CDK4 was shown to play an important role, are adipocytes. In this cell type, insulin activates the cyclin D3-CDK4 complex which subsequently phosphorylates IRS2. This generates a positive feedback loop promoting insulin signaling (Lagarrigue et al., 2016). In the same study, Lagarrigue et al. showed that CDK4-deficient mice display decreased lipogenesis and increased lipolysis in white adipose tissue, indicating that CDK4 is essential for promoting anabolic metabolism in adipocytes (Lagarrigue et al., 2016).

Another cell cycle kinase shown to play an important role in insulin-generating pancreatic β-cells is CDK2 (the CDK responsible for S-phase entry and progression in mammals). In mice, pancreas-specific loss of CDK2 is associated with impaired glucose tolerance and defects in glucose-stimulated insulin secretion (Kim et al., 2017). Moreover, CDK2-deficient mice exhibit a reduced β-cell proliferation when faced with overnutrition or advancing age, indicating that CDK2 both regulates β-cell mass and β-cell function (Kim et al., 2017).

In addition to the cell cycle kinases CDK2 and CDK4, also non-cell cycle CDKs have been described to regulate organismal energy homeostasis in both insulin secreting cells and target cells. For example, CDK5 controls insulin secretion in pancreatic β-cells by phosphorylating and decreasing the activity of the L-type voltage-dependent Ca^2+^ channel (L-VDCC). Knockdown of CDK5 resulted in an enhanced insulin secretion under high glucose conditions suggesting that CDK5 exhibits a negative effect on insulin secretion in β-cells (Wei et al., 2005). In adipocytes, CDK5 regulates glucose uptake by the glucose transporter GLUT4 by several mechanisms (Okada et al., 2008; Lalioti et al., 2009). Silencing of CDK5 decreased glucose uptake by adipocytes indicating that CDK5 positively influences glucose uptake in this cell type (Lalioti et al., 2009). Another non-cell cycle CDK shown to be involved in insulin signaling of adipocytes is CDK8. In the absence of insulin, CDK8-CycC inhibits lipogenesis by phosphorylating SREBP-1c, promoting its degradation. This pathway is conserved from flies to mammals and seems to function completely independently of cell proliferation (Zhao et al., 2012).

## Summary and outlook

The tight coordination between metabolism, growth and proliferation is becoming increasingly clear not only in cancer cells, but across different proliferating cell types in eukaryotic organisms. CDKs play an important role in coordinating metabolism with the cell cycle and ensuring that the carbon and energy demands of proliferating cells are met. Additionally, the G1 CDKs and non-cell cycle CDKs are important for the control of metabolism and energy homeostasis on organismal level, even in non-proliferating cells.

While it is clear that CDKs are involved in the regulation of carbon and energy metabolism across different cell types and species, data is not sufficiently dense yet to judge whether there are general patterns of metabolic regulation that are conserved. Likewise, it is still very much unclear which metabolic fluxes are actually needed to drive each cell cycle phase, and in which phase energy consumption is the highest. This may also be species and tissue dependent, i.e., different cell types may have different strategies to supply the appropriate amount of energy and anabolic precursors in a timely manner.

To address these challenging questions and to solve the roles of different CDKs in regulating metabolism in health and disease *in vivo*, carefully designed studies linking biochemical analysis to cellular and whole animal studies are needed. For obvious practical reasons, many CDK targets screens and investigations have been completed *in vitro* in nutrient environments that do not resemble the *in vivo* situation very well. However, as shown for carbohydrate storage in yeast, the coordination of carbon and energy metabolism with proliferation can be most important when resources are scarce or subject to strong fluctuations (Ewald et al., 2016; Zhao et al., 2016). Nutrient limitation is a constant threat to unicellular organisms (Smets et al., 2010), but can also occur in poorly vascularized solid tumors (Vander Heiden and DeBerardinis, 2017), or in tissues with strongly fluctuating energy requirement such as muscles.

In summary, among the many roles of the cyclin-dependent kinases, regulating carbon and energy metabolism is becoming more and more prominent. Further uncovering this interesting role of CDKs is not only essential for basic cell cycle biology, but may open new avenues for treatment of cancers with overactive CDKs.

## Author contributions

JE designed the topic of the review. MS and JE wrote the manuscript. Both authors read and approved the final version.

### Conflict of interest statement

The authors declare that the research was conducted in the absence of any commercial or financial relationships that could be construed as a potential conflict of interest. The reviewer DS and MS and handling Editor declared their shared affiliation.
