# Supplementary material for: Fueling the Cycle: CDKs in Carbon and Energy Metabolism
Source: Front Cell Dev Biol. 2018 Aug 17;6:93. doi: 10.3389/fcell.2018.00093 (PMC6107797; doi:10.3389/fcell.2018.00093)
Supplement: Supplementary file 1 [file Table_1.DOCX]

Supplementary Table 1: CDK targets involved in carbon and energy metabolism

| Publication | Organism | Cyclin-CDK | Metabolic Target | Cell cycle dependent? | Metabolic Effect |
| --- | --- | --- | --- | --- | --- |
| Direct phosphorylation of metabolic enzymes | | | | | |
| (Santos-Rosa et al., 2005) | *Saccharomyces cerevisiae* | CDK1 | Pah1 (alias Smp2) | Yes | nuclear membrane growth |
| (Kurat et al., 2009) | *Saccharomyces cerevisiae* | CDK1 | Tgl4 | Yes | activation of lipolysis at G1/S transition |
| (Wang et al., 2014) | *Homo sapiens* /MCF-10A cells | cyclin B1-CDK1 | CI subunits of the respiratory chain | Yes | increase of mitochondrial respiration |
| (Ewald et al., 2016) | *Saccharomyces cerevisiae* | CDK1 | Nth1 | Yes | liquidation of trehalose /increase of internal glucose supply |
| (Zhao et al., 2016) | *Saccharomyces cerevisiae* | CDK1 | Nth1, Gph1 | Yes | liquidation of storage carbohydrates /increase of internal glucose supply |
| (Harashima et al., 2016) | *Arabidopsis thaliana* | CDKA;1 | mMDH1, ALDH7B4, pfkB-like kinase, IMD1 | Unclear | not determined |
| (Wang et al., 2017) | *Homo sapiens* /T-ALL cell lines | cyclin D3-CDK6 | PFK1, PKM2 | Unclear | reprogramming metabolism towards PPP and serine pathway/ detox ROS |
| Indirect regulation of metabolism | | | | | |
| (Wang et al., 2006) | *Mus musculus* | cyclin D-CDK | NRF1 | Unclear | inhibition of mitobiogenesis |
| (Taguchi et al., 2007) | *Homo sapiens* /HeLa cells | cyclin B-CDK1 | Drp1 | Yes | mitochondrial fission |
| (Icreverzi et al., 2012) | *Drosophila melanogaster* | cyclin D-CDK4 | NRF-1 targets | Unclear | regulation of mitobiogenesis |
| (Harbauer et al., 2014) | *Saccharomyces cerevisiae* | Clb3- CDK1 | Tom6 | Yes | increase in mitochondrial respiration |
| (Lopez-Mejia et al., 2017) | *Mus musculus* /MEFs  *Mus musculus* /C57BL6 | CDK4 | AMPKα2 | Unclear | upregulation of glycolysis, inhibition of fatty acid oxidation |
| Regulation of organismal energy metabolism | | | | | |
| (Wei et al., 2005) | *Mus musculus* /Min6 cells | CDK5 | L-VDCC | No | regulation of insulin secretion |
| (Okada et al., 2008) | *Mus musculus* /3T3-L1 adipocytes | CDK5 | TC10α | No | regulation of actin polymerization and GLUT4 translocation |
| (Lalioti et al., 2009) | *Mus musculus* /3T3-L1 adipocytes | CDK5 | E-Syt1 | No | regulation of glucose uptake in adipocytes |
| (Annicotte et al., 2009) | *Mus musculus* /Min6 cells | CDK4  (-pRb-E2F) | Kir6.2 | Unclear | insulin secretion |
| (Zhao et al., 2012) | *Drosophila melanogaster*  mammalian hepatocytes  *Mus musculus*/ C57BL6 | CDK8-CycC | SREBP-1c | Unclear | inhibition of de novo lipogenesis |
| (Lee et al., 2014) | *H. sapiens* /U-2OS cells  *Mus musculus* /C57BL6 | cyclin D1-CDK4 | GCN5 | No | suppression of hepatic gluconeogenesis |
| (Lagarrigue et al., 2016) | *Mus musculus* /C57BL6 | cyclin D3-CDK4 | IRS2 | Unclear | maintenance of insulin signalling in adipocytes |
| (Kim et al., 2017) | *Mus musculus* | CDK2 | FOXO1 | Unclear | regulation of β-cell mass and β-cell function |
